# Objective Physiological Measurements but Not Subjective Reports Moderate the Effect of Hunger on Choice Behavior

**DOI:** 10.3389/fpsyg.2018.00750

**Published:** 2018-05-23

**Authors:** Maytal Shabat-Simon, Anastasia Shuster, Tal Sela, Dino J. Levy

**Affiliations:** ^1^Coller School of Management, Tel Aviv University, Tel Aviv, Israel; ^2^Ohalo College, Katzrin, Israel; ^3^Sagol School of Neuroscience, Tel Aviv University, Tel Aviv, Israel

**Keywords:** physiological state, hunger, risk preferences, ultimatum game, choice consistency, alpha-amylase

## Abstract

Hunger is a powerful driver of human behavior, and is therefore of great interest to the study of psychology, economics, and consumer behavior. Assessing hunger levels in experiments is often biased, when using self-report methods, or complex, when using blood tests. We propose a novel way of objectively measuring subjects’ levels of hunger by identifying levels of alpha-amylase (AA) enzyme in their saliva samples. We used this measure to uncover the effect of hunger on different types of choice behaviors. We found that hunger increases risk-seeking behavior in a lottery-choice task, modifies levels of vindictiveness in a social decision-making task, but does not have a detectible effect on economic inconsistency in a budget-set choice task. Importantly, these findings were moderated by AA levels and not by self-report measures. We demonstrate the effects hunger has on choice behavior and the problematic nature of subjective measures of physiological states, and propose to use reliable and valid biologically based methods to overcome these problems.

## Introduction

The feeling of hunger is a ubiquitous phenomenon. Every day we face the cycle of hunger when meal time is approaching and satiation following food consumption. Hunger is affecting almost every human behavior (Loewenstein, 1996), ranging from our internal physiology, through our moods, to different aspects in the process of decision-making. For example, gastric ghrelin production, which is high during hunger, is believed to regulate both food intake (Sarker et al., 2013) and induces adrenocorticotrophin hormone (ACTH) secretion (Kluge et al., 2011), which in turn increases cortisol release and can promote anxiety-like responses (Chuang and Zigman, 2010). Hunger motivates food seeking behavior and food consumption (Nederkoorn et al., 2009). Hunger also induces an approach bias, is associated with an impairment of response inhibition, and affects attention allocation in human subjects (Loeber et al., 2013). Moreover, eating in the absence of hunger is considered an index of disinhibited eating (Francis et al., 2007) and higher impulsivity (Hou et al., 2011; Farrow, 2012).

The hunger cycle has a considerable effect on decision-making. For example, the percentage of favorable rulings by judges drops gradually from ≈65% to nearly zero before a food break and return abruptly to ≈65% afterward (Danziger et al., 2011). This demonstrates that hunger levels presumably affect how judges decide to rule. However, the full effect of changing hunger levels on decision-making yielded somewhat contradicting results. Our aim in the current study is to clarify these inconsistencies using both objective (physiological markers) and subjective (self-reports) measurements of hunger levels, and conduct several behavioral economic tasks within the same individual.

The fundamental finding in previous studies is that the value of a deprived reward, such as food when hungry, is increased in animals (Herrnstein and Loveland, 1974; Epstein et al., 2003; Raynor and Epstein, 2003; Pompilio et al., 2006; Aw et al., 2009) as well as in humans (Hill and McCutcheon, 1975; Spiegel et al., 1989; Drobes et al., 2001; Epstein et al., 2003). Food deprivation also increased the activity in value-related brain areas following presentation of high caloric foods (Siep et al., 2009). Furthermore, a previous study showed that delayed choices were influenced by current hunger as well as future hunger (Read and van Leeuwen, 1998). Hungry subjects chose more unhealthy snacks than did non-hungry subjects, and they chose more unhealthy snacks for immediate consumption than for delayed consumption. However, the study did not directly measure hunger levels for each subject but rather examined all subjects at two time points (separated by 1 week) during the day (after lunch and late afternoon), assuming similar hunger levels for all subjects. Another study demonstrated the link between energy depletion and reduced self-control, and found hunger to promote aggressive behavior (DeWall et al., 2011).

Hunger has also implications on consumer behavior, such that individuals tend to purchase more products (Nisbett and Kanouse, 1969; Mela et al., 1996; Tal and Wansink, 2013) and products higher in caloric content (Tal and Wansink, 2013) when hungry compared to when they are satiated. In addition, financial dissatisfaction results in an increased desire for food energy (Briers and Laporte, 2013). In the social decision-making domain, hungry subjects donated less to charity and to other players in a “give-some game” than did their sated peers, and they ate more chocolate M&Ms after imagining winning 25 than after imagining winning 25,000 (Briers et al., 2006).

It seems that hunger affects risk preferences as well, but the exact nature of this effect is still under debate in the literature (Houston, 1991; Mcnamara and Houston, 1992; Bateson and Kacelnik, 1996; Bednekoff, 1996; Smallwood, 1996; Bateson, 2002; Stephens, 2008). On the one hand, there are studies demonstrating an increase in risk-seeking when an organism is hungry compared to satiated. The idea is that in situations of extreme hunger, which can lead to death, the organism must become risk-seeking and look for food at the expense of increased danger. However, others show the opposite effect and reasoning (Mcnamara and Houston, 1992; McNamara, 1996; Kacelnik and Bateson, 1997; Stephens, 2008). For example, studies of Muslim stock markets indicate that overall market volatility declines sharply during the month of Ramadan, when people fast throughout the day for a month (Seyyed et al., 2005; Bialkowski et al., 2009).

The question that arises is then, why are the effects of hunger on decision making not consistent? We suggest that part of the reason is that most previous studies that examined the effect of hunger on decision-making in humans used subjective reports rather than objective physiological measurements to estimate subjects’ actual level of hunger. The use of questionnaires for estimating hunger levels is, by definition, subjective, and tends to bare various biases. First of all, when using self-report questionnaires, there is a strong assumption that subjects adhere to the experimental manipulation, which usually instructs subjects to refrain from eating for a specific amount of time. Second, it is well documented that subjective reports, such as the visual analog scale (VAS) that is prevalent in hunger studies, can be biased, inaccurate, sensitive to demand effects, and influenced by the social desirability bias (Fisher, 1993; Neeley and Cronley, 2004). Hence, previously reported effects could cause unwarranted theoretical or practical conclusions about consumers’ psychological traits (Campbell, 1950; Peltier and Walsh, 1990), purchase motivations (Levy, 1981), attitudes, intentions, and behaviors (Mensch and Kandel, 1988; Peltier and Walsh, 1990; Podsakoff et al., 2003), as well as contradicting conclusions regarding the effect of hunger levels on risk preferences (Levy et al., 2013).

In addition, when using the VAS there is a strong assumption that hunger levels increase linearly. Moreover, usually scientists treat the VAS as a “true” quantitative ratio scale, which is inaccurate. Assuming a ratio scale to scores that do not behave in such a manner may lead to a biased estimation of the measured variable (Narens, 1981; Alper, 1985; Luce, 2001), and thus may lead to inaccurate (and in some cases opposite) conclusions regarding the measured effect. Even more problematic is that some studies (e.g., Briers et al., 2006) rely solely on the experimental manipulation and examine average group effects, ignoring the large variability in hunger levels across subjects.

Therefore, there is a need for an objective and unbiased measurement in order to better understand the effect that different hunger levels have on decision-making, and it needs to be on a subject-specific basis. The most straightforward method is to rely on physiological measurements, which are objective and are very hard to control or intentionally bias without active external interference. A common method for estimating hunger levels is to draw blood from subjects at several time points during the experiment and examine glucose levels. However, using glucose levels does not always give a reliable or consistent result for hunger levels (for a review see de Graaf et al., 2004). Other, more reliable signals of hunger levels are ghrelin – both for short and long term levels (Tschop et al., 2000; Cummings et al., 2001; Nakazato et al., 2001; Wren et al., 2001; Druce et al., 2005; Klok et al., 2007) and leptin – for long-term energy balance (Sawchenko, 1998; Klok et al., 2007). Although using blood samples to estimate subjects’ hunger levels is relatively accurate when measuring ghrelin levels, there are several drawbacks to applying this method in field studies or to use in marketing research in the industry. Drawing blood is complicated, invasive, expensive, and requires expertise and extensive approvals. Hence, we propose to use saliva samples instead of blood samples in order to estimate objective physiological levels of hunger in humans. Saliva samples are easy and cheap to gather, do not require expert personnel, easily conducted anywhere, can be analyzed relatively fast, and are far less invasive than blood draws. However, ghrelin and leptin are not well detected in saliva; therefore, an alternative marker is needed.

In the current paper, we are using a physiological marker that was only recently associated with hunger levels – alpha-amylase (AA) (Harthoorn and Dransfield, 2008). Salivary AA is one of the most important enzymes in saliva that catalyzes the hydrolysis of starch and glycogen (Yang et al., 2015), and is involved in defense against pathogens (mainly food-born) (Petrakova et al., 2015). AA secretion increases as a result of two main events: one, AA has a pronounced and distinct diurnal rhythm with a strong drop in activity in the first hour after awakening, followed by a steady increase toward the evening (Nater et al., 2007). Note that this diurnal cycle is not related to food consumption or hunger levels. Second, AA secretion increases after food consumption. AA is secreted into the oral cavity from the acini cells of the parotid gland (O’Donnell et al., 2010), which is innervated by efferent sympathetic nerves (Vanstegeren et al., 2006). Thus, AA secretion following food intake is a direct response to the activity of the sympathetic nervous system (Nater et al., 2006; Harthoorn and Dransfield, 2008; Jayasinghe et al., 2014). Previous studies demonstrated the link between salivary AA and food intake, especially its role in metabolism of carbohydrates (Squires, 1953; Jayasinghe et al., 2014). The increase in salivary AA following food consumption appears to be related to the nutrient content of the consumed food (Squires, 1953; Mejean et al., 2015), as well as to the consumed amount and, importantly, to perceived fullness, i.e., not being hungry (Harthoorn, 2008; Harthoorn et al., 2009). The most straightforward evidence for the link between salivary AA and hunger comes from a study that explicitly examined the relationship between food intake, self-reported levels of satiety and hunger, and levels of salivary AA. The authors found a positive correlation between AA increase after a meal and satiety and fullness, alongside a negative correlation with hunger and the desire to eat. Another study (Harthoorn, 2008) reported two relevant findings: (1) a negative correlation between the amount of food consumed and the level of AA before consumption, in line with the assumption that hungrier subjects would eat more and (2) a positive correlation between the amount of food consumed and the level of AA after consumption, suggesting that subjects who ate more felt less hungry.

Therefore, based on these previous studies and extensive preliminary examinations conducted in our lab, we chose to use salivary AA as our indicator for subjects’ shift from hunger to not-being hungry following food intake. Importantly, an objective subject-specific measurement in each session has the additional benefit of annulling individual differences in baseline hunger levels due to differences in food consumption on the day before the experiment.

To conclude, in the current study our aim was twofold: First, to examine how a change in hunger levels affect choices in different tasks. Second and more importantly, we directly contrasted the effect of objective (AA levels) with subjective (VAS scores) measurements of an internal human state and examined which of them could reliably explain some of the behavioral changes in choices. In order to do so, subjects came to the lab twice – once hungry and once satiated. We define hunger as the physiological state after 12 h of fasting, corresponding to skipping one’s breakfast. In each session, subjects completed three choice tasks and we estimated their objective and subjective levels of hunger by measuring AA levels in collected saliva samples as well as collecting their subjective reports on a VAS.

We had several hypotheses. First, we examined if we would replicate previous findings (Levy et al., 2013) that, on average, less hungry subjects (compared to hungry subjects) are less risk averse. Second, that hungry subjects will tend to reject more (compared to when being less hungry) unfair offers in the ultimatum game. Third, that hungry subjects will be less consistent in their choices. Fourth, and most importantly, that the behavioral effects will be moderated by the objective, but not the subjective, measurements of hunger.

We present here data that corroborate our first two hypotheses but not the third one. Additionally, we clearly demonstrate that only objective measurements, but not subjective reports of hunger, moderate the effect on subjects’ choices, thereby corroborating our forth hypothesis. We show that hunger levels affect subjects’ choices and that when estimating the effects of internal state, it is crucial to use a physiological objective measurement because it is more reliable, accurate, and less biased than subjective measurements based on self-report.

## Materials and Methods

### Participants

A total of 57 participants (28 women) were enrolled in this study and completed two behavioral sessions. All of the participants were students at Tel Aviv University. None of the participants reported a history of eating disorders. All participants gave written informed consent. All procedures were in compliance with the safety guidelines for behavioral research and were approved by the University Committee on Activities Involving Human Participants at our university. Out of the 57 participants, 6 participants failed to deliver saliva samples, 2 participants failed to fast for 12 h prior to the behavioral sessions, and for 3 participants parametric risk parameters could not be accurately estimated due to random choices. All these participants were discarded from all further analyses. All data reported here are based on the remaining 46 participants (21 women).

### General Procedure

**FIGURE 1:**
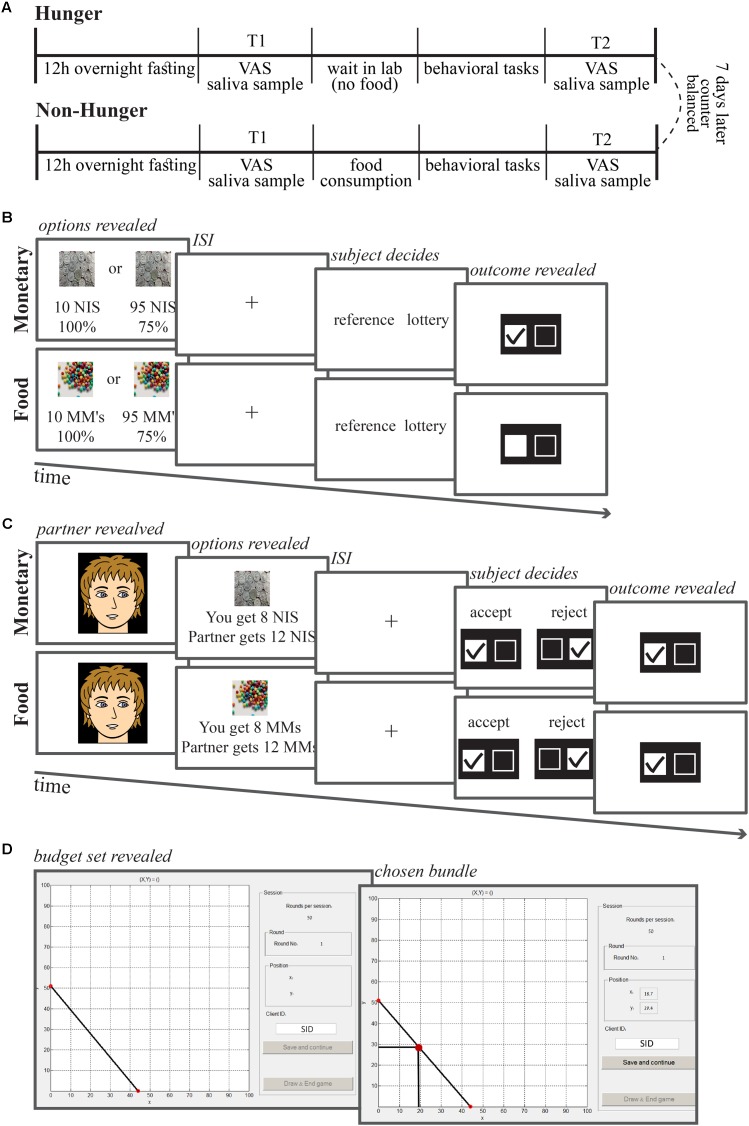
Session and task timelines. **(A)** Each subject came to the lab for two sessions, 1 week apart. To both sessions subjects came after 12 h fasting (only water allowed). Upon arrival (10:00 AM) subjects delivered a saliva sample and stated their subjective level of hunger on an analog scale. Afterward they were either given breakfast or not, and completed three computerized behavioral tasks. Then, at 11:15 AM, they delivered the second sample of saliva and indicated subjective hunger levels. **(B)** Risk task trial timeline. On each trial, subjects were asked to choose between a safe option (10 NIS or 10 chocolate M&Ms) and a lottery option (some probability to win some amount of chocolates or NIS). **(C)** Ultimatum game task trial timeline. On each trial, subjects were presented with a photograph of a partner, and then the offer from that partner. The subjects were asked to either accept or reject the offer. **(D)** Economic inconsistency task trial timeline. On each trial, subjects were presented with a budget set of two lotteries with equal probabilities (50–50% chance of winning) corresponding to the *X*- and *Y*-axes of the graph. Subjects were asked to allocate an endowment of tokens between these two lotteries. ISI, inter-stimulus interval.

The study had a within-subject design with two sessions, *Hunger* and *Non-Hunger* (**Figure 1A**). Subjects were informed that the study examines the influence of physiological state on decision-making. In both sessions, subjects were asked to refrain from eating for 12 h before arriving to the laboratory. During these 12 h, subjects were allowed to drink only water and avoid any sweet beverages, coffee, or cigarettes. We started each session at 10:00 AM. We asked subjects to report what they ate the day before and the time at which their last meal was. We used this information to ensure subjects’ compliance with the fast. In each session, subjects gave a saliva sample and a self-report of hunger at two time-points (T1 and T2) and conducted behavioral choice tasks between these time points. We took the first sample (T1) upon subjects’ arrival and a second sample (T2) 75 min later (at 11:15 AM) after the end of the behavioral tasks.

In the *Non-Hunger* session, immediately after providing the first saliva sample (T1), participants were asked to eat a controlled breakfast that we provided (two cheese/tuna-salad/omelet sandwiches). All of the subjects ate at least one sandwich, 15% ate more. They could eat as much as they wanted for 15 min and up to two sandwiches. Note that the continuous nature of the AA measure allows us to examine the influence of hunger on behavior even when subjects are not matched for the amount of food they consume, since subjects that ate less are expected to have a smaller increase in AA, which would correspond to a predicted smaller change in behavior. Thereafter, subjects conducted the behavioral tasks and at the end of the behavioral tasks we collected the second saliva sample (T2).

In the *Hunger* session, subjects did not have any breakfast and after delivering the first saliva sample (T1) they waited in the lab for 15 min to resemble the time frame as in the *Non-Hunger* condition, and then conducted the behavioral tasks. After completing them, they delivered their second saliva sample (T2). The order of the two sessions was counterbalanced by randomly assigning subjects to their first session (*Non-Hunger* and *Hunger*).

Before each saliva sample, we also collected a subjective report of hunger levels using a standard VAS. Subjects reported their current hunger levels by marking a point on a scale that had the instruction “Please mark on the scale the level of your current hunger.” The scale stretched from “not at all hungry” to “very hungry.” Subjects received 240 NIS show up fee (∼$60) after the completion of both sessions (4 h in total) plus any other earnings based on their choices.

### Behavioral Choice Tasks

In each session, between the two time-points of assessing objective and subjective hunger levels (T1 and T2), subjects performed three behavioral choice tasks: (1) Assessing risk preferences for money and food – risk task. (2) Assessing prosocial behavior for money and food – ultimatum game. (3) Assessing choice consistency over money. The order in which subjects performed the tasks was counterbalanced across subjects and across sessions. The behavioral part of each session lasted approximately 1 h. Importantly, we informed subjects in advance that after the end of the session, they would be asked to remain in the laboratory for an additional 45 min during which the only food they would have access to is the food realized from one trial from each task selected randomly at the end of the experiment.

#### Risk Task

In order to assess individual risk preferences as a function of hunger levels, subjects conducted a risk choice task over monetary and food (M&Ms – chocolate candies) rewards. As can be seen in **Figure 1B**, on each trial, subjects made a choice between a certain small reward (the *reference* option) and a stated probability of either winning a larger amount of the same reward (money or food) or getting nothing (the *lottery* option). In all trials the *reference* option was fixed (10 NIS or 10 chocolate candies). Five different values for the *lottery* option for each reward type (10–95 NIS and 10–95 candies) were fully crossed with five winning probabilities (13, 22, 38, 50, and 75%) yielding 25 unique lottery options for each reward type. Each unique choice option was presented four times throughout the task for a total of 100 choices for each reward type.

On each trial, two options were presented on a computer screen for 2 s. This was followed by a white cross in the middle of a black screen, indicating subjects to choose the option they preferred, by pressing one of two buttons on a computer mouse. Subjects had 1.5 s to choose followed by a feedback for 0.5 s that indicated subject’s choice on the current trial (left or right). The next trial then followed immediately. Note that the feedback was not regarding the consequence of their choice but was only a visual checkmark representing what subjects chose on that trial (lottery or reference option) without any real feedback of the outcome of that trial. Failing to make a choice within the given time resulted in an error signal during the feedback interval. We omitted all missed trials from the analysis.

We used the choice data to estimate the rate at which the subjective value grows as a function of objective value for each of the reward types for each subject under different internal states. To infer the value functions from these data we use standard tools from behavioral economics (Wu and Gonzalez, 1996; Holt and Laury, 2002, 2005) that were employed previously for money (Levy et al., 2010) and then later modified for food (Levy and Glimcher, 2011; Levy et al., 2013). We used a standard logistic function that relates subjects’ choices to the expected value (EV) of each lottery. We then added the AA levels and VAS scores (as well as their interactions) as additional regressor to the analysis. Using this approach, we can ask how do hunger levels affect subjects’ choices over money and food in a risk task above and beyond the EV of the lottery and if it moderates the effect of EV on choice. Our baseline hypothesis was that, on average, subjects will become less risk averse (more risk-seeking) when hungry, which is compatible with some studies in animals (Bateson and Kacelnik, 1996; Bednekoff, 1996; Smallwood, 1996; Kacelnik and Bateson, 1997; Bateson, 2002; Stephens, 2008) and in humans (Symmonds et al., 2010; Levy et al., 2013). Additionally, we hypothesized that objective but not subjective measurements will moderate the effect of EV on risky choices.

#### Ultimatum Game

Each subject conducted 10 consecutive trials of a standard ultimatum game as the *responder* (Guth et al., 1982), consisting of five trials for each reward type. As can be seen in **Figure 1C**, on each trial, subjects had to choose to accept or reject an offer from a *proposer* out of a total of 20 NIS or 20 candies. On each trial before the actual offer, a picture of a neutral face (taken from the FEI Face Database, http://fei.edu.br/tildecet/facedatabase.html) representing the *proposer* (randomly chosen from a set of 10 proposers – 5 females and 5 males) was presented for an unlimited time until the subject pressed a button. This was followed by the offer, which could take five different amounts – 2, 4, 6, 8, or 10 NIS or candies, out of a total of 20 NIS or 20 candies. The offer was followed by a fixation cross for 500 ms, and thereafter, subjects chose whether to reject or accept the offer using the mouse buttons. Subjects knew that if they accepted the offer they could receive the amount that was offered to them while if they rejected the offer then they, as well as the *proposer*, would get nothing. Subjects also knew that at the end of the experiment one trial from each reward type would be randomly picked to account for real payoff. Subjects were told that the offers were submitted by real people; however, they were predefined and fixed for all subjects, so that all subjects faced the exact same offers. At the end of the experiment, we debriefed the subjects to ensure they did not suspect the manipulation. Subjects’ compliance with the task as well as their responses in the debriefing indicate they believed the manipulation.

#### Economic Inconsistency

We estimated subjects economic consistency by testing the generalized axiom of revealed preference (GARP) (Samuelson, 1938; Houthakker, 1950). The task was similar to the one used and described in Choi et al. (2007). As can be seen in **Figure 1D**, on each trial, subjects were presented with a budget-set using a graphical interface. Subjects were requested to allocate an endowment of tokens (using the mouse curser) between two lotteries with equal probabilities (50–50% chance of winning) corresponding to the *X*- and *Y*-axes on a two-dimensional graph. Each subject completed 50 trials. Allocating the tokens to a boundary solution (to one of the edges of the budget set {(0,*Y*) or (*X*,0)} here there is a 50% chance that the subject will not get any money) represents risk-seeking preferences, while purchasing the same amount from both accounts represents extreme risk aversion. A risk neutral choice is considered to be the point on the budget set that results in the highest EV possible for that budget set. We calculated EV in the standard way: *P_x_* ^∗^ *X* + *P_y_* ^∗^ *Y.*

Where {*P_x_*_,_ *P_y_*} are the prices for lotteries *X* and *Y*, respectively, and {*X*, *Y*} are the amount of tokens of lotteries *X* and *Y*, respectively. Intermediate solutions represent other preferences, which do not fall into one of these three pure types. The prices of buying the lotteries (as indicated by the slope of the lines) were randomly chosen across trials. Note that there was no feedback regarding the outcome of that trial. Subjects were told that one trial will be randomly chosen at the end of the experiment and will be played out for real monetary reward. Based on subjects’ allocations, we estimated the level of economic inconsistency (GARP violations) for each subject using three well-known indices taken from the economic literature – Afriat index (Afriat, 1973), Varian index (Varian, 1990), and Houtman–Maks index (Houtman and Maks, 1985).

### Saliva Samples Collection and Analysis of AA Levels

Subjects provided a total of four saliva samples (two in each session). We collected the samples using SALIVETTE saliva collecting tube. Each participant provided a baseline sample (T1) upon arrival at the lab at 10:00 AM, and a second one at 11:15 AM (T2), after completion of the behavioral tasks. For each saliva sample collection, subjects were requested to insert a small sterile swab into their mouth for 120 s, and then place it into a clean sterile tube. We placed the tubes in a centrifuge for 2 min at 1000 ×*g*, placed the clear saliva sample into a 1.5-ml sterile tube, and froze them immediately at -20°C. For the analysis of AA, we used a commercial ELISA kit from EUROIMMUN (96 wells). We diluted the samples 1:201 with sample buffer (e.g., 5 μl sample to 1.0 ml sample buffer), applied 20 μl per well from each sample, calibrators and control. We then added 100 μl of enzyme conjugate and 100 μl of antiserum to each well. Thereafter, we covered the wells and incubated the plate for 60 min at room temperature on an orbital shaker (400 rpm). After the incubation, we washed the plate three times (300 μl of washing buffer per well), added 100 μl of substrate to each well (to produce color), incubated the plate for an additional 15 min at room temperature, and finally added a stop solution (otherwise all samples would yield the same optical density/color). We then evaluated AA levels in the samples using photometric measurements (450 nm). To quantify AA levels in our samples, we measured the color that was devolved in each well.

### Statistical Analysis

#### AA and VAS Measurements

To examine our basic manipulation of hunger, we measured both the AA levels and VAS scores in two time-points (T1 and T2) in each session (*Hunger* and *Non-Hunger*) and examined the change between the two time-points. To eliminate the effect of outliers and the large differences between subjects in baseline levels, we mean-centered the data (within subjects’ state mean) and transformed the AA levels into a logarithmic scale.

Importantly, in order to take into consideration that AA levels increase not only due to food intake but also because of its diurnal cycle, we generated a subject-specific index for the change in AA levels of each session by taking the difference in AA levels between T1 and T2 of that session. We used the AA difference of each session as a subject-specific measure of the shift from being hungry to not (or less) hungry, and regressed them with subjects’ choices in our different behavioral tasks. Note that larger differences in AA levels between the time-points would predict a greater difference in behavior.

We flipped the direction of the VAS scale to be aligned with AA levels. That is, a higher VAS score indicates less hunger.

#### General Regression Approach

In order to examine the effect of AA levels and VAS scores on behavior, we included these scores as independent variables in all analyses. This method allowed us to directly compare AA levels and VAS scores effects on behavior.

In the risk task we measured subjects’ preferences toward risk for both monetary and food rewards. Subjects conducted the exact risk task for both rewards. We fitted a logistic regression model separately for each reward type that included EV, *Condition* (*Hunger*/*Non-Hunger*), AA levels, and VAS scores, we well as their interactions, as the independent variables. Subjects’ choices to accept the lottery or the certain amount (the *reference* option) served as the dependent variable. AA levels and VAS scores were mean-centered before they were entered into the analysis. Note that there were two AA/VAS scores for each subject – the score from the *Hunger* condition and the score from the *Non-Hunger* condition.

In the ultimatum game subjects made a series of choices to either accept or reject an offer from an individual. The offer was some amount of money or pieces of chocolate candies out of a total of 20 NIS/20 candies. We measured for each subject and for each reward type whether they decided to accept or reject the different amounts offered. For the statistical analyses, we generated a fairness index (FI) by separating the offers into fair offers (i.e., offers of 8 or 10 NIS/candies) and unfair offers (i.e., offers of 2 or 4 NIS/candies). Similar to the risk task, we fitted a logistic regression separately for each reward type with FI, *Condition* (*Hunger*/*Non-Hunger*), AA levels, VAS scores, as well as their interactions as the independent variables. Subjects’ choices to accept/reject the offers served as the dependent variable. We defined for each subject their sensitivity to fairness by subtracting the propensity to accept unfair offers from the propensity to accept fair offers. A large difference between these propensities would indicate a subject who is highly sensitive to fairness, since the fairness of the offer greatly affects her behavior.

#### Correlation Analysis

We calculated Pearson correlations between AA and VAS measurements in order to examine reliability and validity for these measurements within a day and across time points. We also calculated Pearson correlations across tasks, in order to examine task consistency across time points and between different aspects of decision making behavior.

We used Stata v14.2 to analyze the data. We calculated robust SE for all of the regression models reported here using Stata’s clustered sandwich estimator [i.e., vce(cluster clustvar) option in Stata]. To probe interactions, we evaluated margins of responses. This method allows us to estimate and understand interactions using Pick-a-Point procedure [i.e., analysis of simple slopes (Rogosa, 1980; Cohen et al., 2013; Preacher et al., 2016)].

## Results

### AA and VAS Measurements (Objective Measurement vs. Subjective Report)

We first examined if our basic manipulation of *Hunger*/*Non-Hunger* worked across sessions by analyzing the objective (salivary AA levels) and subjective (VAS scores) hunger levels in both sessions.

As can be seen in **Table 1**, a repeated-measures ANOVA revealed significant main effects for Time (T2 vs. T1) and Condition (*Hunger* vs. *Non-Hunger*) for both AA and VAS (all *p* < 0.01). More importantly, the interaction between Time and Condition was significant for both AA and VAS (both *p* < 0.0001). *Post hoc* paired-sample *t*-tests revealed a significant difference between T1 and T2 in both conditions (*Hunger* and *Non-Hunger*), for AA and VAS (all *p* < 0.01, **Table 2**).

**Table 1:** Effect of time (beginning/end of the experimental session) and condition (hunger/non-hunger) on levels of alpha-amylase (AA) and visual analog scale (VAS).

AA				VAS			
Source	*df*	Mean square	*F*(*p*)	Source	*df*	Mean square	*F*(*p*)
Time	1	2.67	90.25^∗∗∗^	Time	1	116.80	50.23^∗∗∗^
Condition	1	0.37	13.51^∗∗^	Condition	1	730.41	177.10^∗∗∗^
Time × Condition	1	0.26	23.33^∗∗∗^	Time × Condition	1	348.98	205.83^∗∗∗^

*^∗^p < 0.05, ^∗∗^p < 0.01, ^∗∗∗^p < 0.0001.*

**Table 2:** Levels of AA and VAS at the beginning (T1) and end (T2) of the experimental session.

Parameters	Mean	STD	SEM	*t*	*df*
**AA**					
T1_Hunger_ vs. T1_Non-Hunger_	-0.01	0.20	0.03	-0.48	45
T2_Hunger_ vs. T2_Non-Hunger_	-0.17	0.19	0.028	-5.87^∗∗∗^	45
T1_Hunger_ vs. T2_Hunger_	-0.17	0.19	0.03	-5.861^∗∗∗^	45
T1_Non-Hunger_ vs. T1_Non-Hunger_	-0.32	0.21	0.03	-10.122^∗∗∗^	45
T2_Hunger_ - T1_Hunger_ vs. T2_Non-Hunger_ - T1_Non-Hunger_	-0.15	0.21	0.03	-4.83^∗∗∗^	45
**VAS**					
T1_Hunger_ vs. T1_Non-Hunger_	-1.23	2.43	0.36	-3.43^∗∗^	45
T2_Hunger_ vs. T2_Non-Hunger_	-6.74	2.39	0.35	-19.11^∗∗∗^	45
T1_Hunger_ vs. T2_Hunger_	1.16	1.12	0.17	7.077^∗∗∗^	45
T1_Non-Hunger_ vs. T1_Non-Hunger_	-4.35	2.6	0.38	-11.326^∗∗∗^	45
T2_Hunger_ - T1_Hunger_ vs. T2_Non-Hunger_ - T1_Non-Hunger_	-5.51	2.6	0.38	-14.347^∗∗∗^	45

*^∗^p < 0.05, ^∗∗^p < 0.01, ^∗∗∗^p < 0.0001.*

As expected by our manipulation, subjects reported that they were hungrier in the *Hunger* session than in the *Non-Hunger* session – VAS scores in the *Hunger* condition were lower overall than in the *Non-Hunger* condition (mean VAS in *Hunger* = 2.16, mean VAS in *Non-Hunger* = 6.03, *t*_45_ = -9.66, *p* < 0.001). Furthermore, subjects reported to be hungrier (lower VAS) at the end of the experiment (T2; *M* = 1.56, *SD* = 1.8) compared to the beginning (T1; *M* = 2.72, *SD* = 1.9) in the *Hunger* session (subjects did not eat for another 80 min between T1 and T2), and less hungry (higher VAS) in T2 (*M* = 8.3, *SD* = 2.15) compared to T1 (*M* = 3.95, *SD* = 2.5) in the *Non-Hunger* condition (after consumption of a sandwich) (*p* < 0.0001).

**FIGURE 2:**
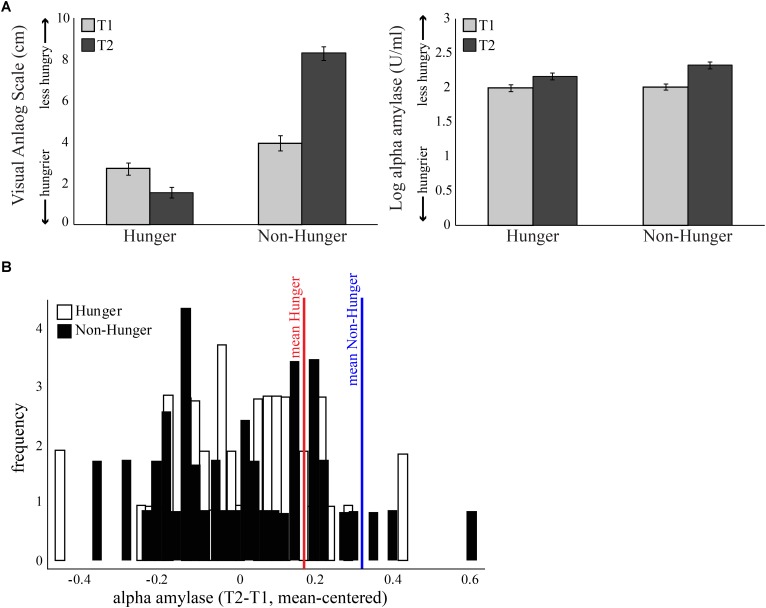
Subjective measure of hunger levels. **(A)** Both objective and subjective measures were given at four time points – at the beginning of each session (*Hunger* session and *Non-Hunger* session; T1) and after completion of behavioral tasks (T2), 1.25 h later. Left: average score of subjective hunger levels provided by a visual analog scale. Right: average concentration of salivary alpha-amylase (AA). Error bars indicate standard error of the mean. **(B)** Subject distribution of the salivary AA levels of each subject, normalized using the baseline levels at the beginning of the session. In the hunger session, the increase in AA is above and beyond the increase caused by the natural diurnal rhythm.

However, a troubling finding regarding the subjective reports appears in the hunger levels at the beginning of the experiment. As can be seen in the left panel of **Figure 2A**, T1 hunger levels reported in the *Hunger* condition are significantly higher (lower VAS) than that of T1 in the *Non-Hunger* condition [difference of -1.23, *t*(45) = -3.43, *p* < 0.01], although subjects were supposed to have similar initial hunger levels (T1 in both sessions is the baseline after 12 h of deprivation). To summarize the VAS results, subjects reported hunger levels that they presumably felt, and/or what they thought is expected of them by the manipulation and/or experimenter. This is our first evidence that subjective reports are possibly not an accurate indication of subjects’ “true” hunger levels, but rather a mixture of hunger levels and demand effects or a desirability bias.

On the other hand, as can be seen in the right panel of **Figure 2A** and **Table 2**, examination of the objective AA levels revealed to be a more promising measurement for an accurate indication of subjects’ hunger levels. As expected, the AA levels were very similar in the beginning of both sessions (difference of -0.01, *p* = 0.63). Furthermore, in the *Hunger* session, there was a small increase in AA levels between T1 (*M* = 1.99, *SD* = 0.34) and T2 (*M* = 2.16, *SD* = 0.34), due to the well-documented diurnal rhythm of the enzyme, which tends to naturally increase throughout the day, not related to hunger levels (Nater et al., 2007). Comparison of T2 in the *Hunger* session (no food at all) and T2 in the *Non-Hunger* session (after subjects ate) should indicate the change in hunger levels between the sessions. Indeed, AA levels in *T2*-*Non-Hunger* (*M* = 2.32, *SD* = 0.32) were significantly higher than in *T2*-*Hunger* (*M* = 2.16, *SD* = 0.34) (*t*_45_ = 5.85, *p* < 0.001), indicating that food consumption increased AA levels above and beyond the diurnal rhythm. Hence, a bigger increase (between T1 and T2) in AA levels in the *Non-Hunger* condition indicates that food made these subjects less hungry, while a small difference in AA levels indicates a hungrier subject. **Figure 2B** describes the across-subjects distribution of the net effect of *Hunger-*/*Non-Hunger*-mediated AA levels in both experimental conditions. Note that there is a shift to the right of the distribution when moving from *Hunger* (*M* = 0.16, *SD* = 0.002) to *Non-Hunger* (*M* = 0.31, *SD* = 0.002).

#### Correlation Analysis: AA and VAS

As shown in **Table 3**, AA is a highly reliable measure that captures hunger levels. We found that AA levels have a high test–retest reliability *within* and *across* days. That is, within a day, the T1–T2 AA levels are highly correlated in both the *Hunger* condition (when subjects arrive to the lab after 12 h fast and do not receive food at all; *r* = 0.84) and in the *Non-Hunger* condition (when subjects arrive to the lab after 12 h fast and receive food; *r* = 0.78). Importantly, we also have great test–retest reliability across days: there is a very high correlation (*r* = 0.808) between AA measures at T1 *Hunger* and T1 *Non-Hunger* (in both cases subjects arrived to the lab after 12 h fast).

**Table 3:** Correlations of subjective (VAS) and objective (AA) hunger measures between sessions (hunger/non-hunger) and time points (T1/T2).

		(1)	(2)	(3)	(4)	(5)	(6)	(7)	(8)
(1)	log(AA) Hunger T1	1							
(2)	log(AA) Hunger T2	0.843^∗∗∗^	1						
(3)	VAS Hunger T1	0.071	0.184	1					
(4)	VAS Hunger T2	0.185	0.166	0.826^∗∗^	1				
(5)	log(AA) Non-Hunger T1	0.808^∗∗∗^	0.744^∗∗∗^	0.084	0.112	1			
(6)	log(AA) Non-Hunger T2	0.737^∗∗∗^	0.840^∗∗∗^	0.027	0.054	0.778^∗∗∗^	1		
(7)	VAS Non-Hunger T1	-0.084	0.070	0.432^∗∗^	0.269	-0.160	0.094	1	
(8)	VAS Non-Hunger T2	-0.170	0.051	0.323^∗^	0.278	-0.186	0.057	0.386^∗∗^	1

*^∗^p < 0.05, ^∗∗^p < 0.01, ^∗∗∗^p < 0.001.*

On the other hand, VAS scores exhibit unstable test–retest reliability. When we look at test–retest consistency within a day we see that in the *Hunger* condition the T1–T2 VAS scores are highly correlated (*r* = 0.82). However, in the *Non-Hunger* condition the T1–T2 VAS scores are only moderately correlated (*r* = 0.39). More importantly, the test–retest consistency across days is weak (*r* = 0.432). Although the latter correlation is significant, in terms of test–retest reliability this is quite low. One would like to find a correlation value of at least 0.7 as a rule-of-thumb to establish proper reliability. Regardless of this threshold, we found that AA test–retest is significantly higher than VAS test–retest (*r*_AA_ = 0.808 vs. *r*_V AS_ = 0.432, *p* = 0.0022, two-tailed, Fisher’s *r*-to-*z* transformation).

As shown in **Table 3**, there is no correlation between AA and VAS measures. This null correlation produces, apparently, a problem with AA because it is possible to claim that AA is not directly related to hunger levels. However, a classical method to validate the construct validity of a new instrument (and to show the tool measures what we claim that it measures) can discriminate between two groups known to differ on the variable of interest [i.e., known-groups validity (Davidson, 2014)]. Hence, based on our results we claim that AA distinguishes well between the groups (i.e., we get robust statistical differences) and we can use it as a valid instrument to differentiate hunger states. In addition, this instrument has very high reliability, within and across days. On the other hand, VAS is highly susceptible to demand effects (VAS scores are only correlated within a day and not between days). Furthermore, reliability sets an upper bound on validity. That is, the correlation between VAS scores and any other variable (a score in any of the tasks) cannot exceed the reliability of that tool, thus hinders the usage of VAS. To conclude, the analysis of basic psychometric properties of AA and VAS measures reveals that AA levels are superior to VAS scores as a method to capture individual differences in hunger. Thus, importantly, we demonstrated here that we have a powerful tool to objectively measure each subject’s hunger levels using saliva samples without the need to depend on inaccurate and biased subjective reports, nor technically demanding and expensive techniques such as blood samples.

### Risk Task

After establishing our objective measure of *Hunger*/*Non-Hunger* levels, we examined how hunger levels influenced subjects’ preferences toward risk for both monetary and food rewards. We first omitted no-response trials from any further analysis – 356 trials out of 18,400 trials from all sessions and reward types. This corresponds to ∼2% in general and an omission of around 0.5% trials from each task (monetary/food) in each condition (*Hunger*/*Non-Hunger*).

**Table 4** shows a significant main effect of EV on choice for both monetary (*B* = 0.12, *p* < 0.0001) and food rewards (*B* = 0.09, *p* < 0.0001), indicating that subjects tended to choose the lottery option more as its EV increased. Importantly, we found a significant interaction between EV and AA levels for choices over monetary rewards (*B* = -0.12, *p* < 0.01), and the same coefficient tendency (albeit did not reach significance, *B* = -0.04, *p* = 0.64) for food choices. In order to breakdown this interaction, we have split our data into five bins as a function of the standard deviation (SDs) around the mean (see the section “General Regression Approach” in the section “Materials and Methods” for more details).

**Table 4:** Effect of expected value (EV) of the lottery, condition (hunger/not-hunger), subjective hunger measure (VAS), and objective hunger measure (AA) on risk preferences.

	Reward type					
	Monetary			Food		
Parameters	*B*	*SE*^a^	*Z*	*B*	*SE*^a^	*Z*
EV	0.12	0.01	12.7^∗∗∗^	0.09	0.01	8.9^∗∗∗^
Condition	0.13	0.2	0.65	0.08	0.21	0.36
Condition × EV	-0.01	0.01	-1.59	-0.01	0.01	-0.59
AA	0.2	0.99	0.2	-0.26	1.11	-0.23
EV × AA	-0.12	0.04	-2.68^∗∗^	-0.04	0.09	-0.46
Condition × AA	0.58	1.08	0.54	1.23	1.15	1.07
Condition × EV × AA	0.12	0.05	2.56^∗^	0.02	0.08	0.24
VAS	0.1	0.21	0.45	-0.12	0.22	-0.54
EV × VAS	-0.01	0.01	-1.42	-0.01	0.01	-0.61
Condition × VAS	0.07	0.21	0.33	0.33	0.25	1.32
Condition × EV × VAS	0.01	0.01	1.18	0.01	0.01	0.95

*^a^Robust Std. Err. (errors clustered by subject); ^∗^p < 0.05, ^∗∗^p < 0.01, ^∗∗∗^p < 0.0001.*

**FIGURE 3:**
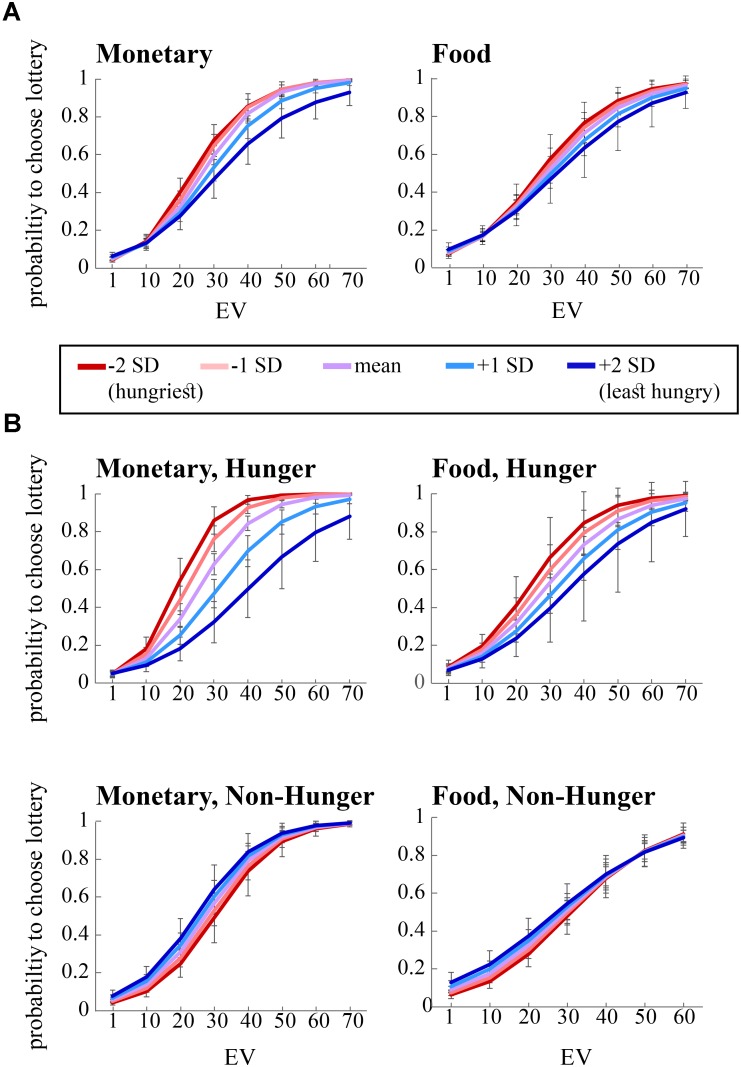
Risky choice is influenced by expected value (EV), condition (*Hunger* or *Non-Hunger*), and objective level of hunger. **(A)** Hungrier subjects (negative standard deviations from the mean of AA, red lines) are more likely to choose the lottery option in the risk task over the safe option, compared to less hungry subjects (positive SDs from the mean of AA, blue lines). **(B)** In the *Non-Hunger* session (bottom panels), no variation in subjects’ behavior was found. In the *Hunger* session (top panels), the hungrier a subject was, the more likely they were to choose the lottery option over the safe option.

As can be seen in the left panel of **Figure 3A**, the significant interaction between EV and AA levels (*B* = 0.12, *p* < 0.05) indicates that, across conditions, the probability to choose the lottery for monetary rewards increases as a function of both EV and AA levels. That is, AA levels moderate the probability to choose the lottery, such that the hungrier subjects (lower levels of AA – negative SDs) were more likely to choose the lottery. Furthermore, we found a significant interaction between EV, AA, and *Condition* for choices over monetary rewards. The right panel of **Figure 3A** shows the same coefficient tendency (albeit did not reach significance, *B* = 0.02, *p* = 0.8) for food choices.

In order to decompose the three-way interaction in the risk task (monetary reward), we fitted two separate regression models – one for the *Hunger* condition and one for the *Non-Hunger* condition. Breaking the three-way interaction by *Condition* revealed that there is a significant two-way interaction only within the *Hunger* condition. That is, we see a main effect for EV for both conditions (*B* = 0.12, *p* < 0.000 and *B* = 0.11, *p* < 0.000 for *Hunger* and *Non-Hunger*, respectively) but we find an EV by AA interaction only in the *Hunger* condition (*B* = -0.08, *p* < 0.05 and *B* = 0.002, n.s. for *Hunger* and *Non-Hunger*, respectively). That is, breaking down the triple interaction revealed that the probability to choose the lottery for monetary rewards increases as a function of EV and AA levels mainly under the *Hunger* condition and not in the *Non-Hunger* condition (**Figure 3B**). Since this is a continues by continues interaction we further decompose it using the different AA levels (-2, -1, 0, 1, and 2 SDs) and calculated the relation between EV and choice propensity as presented in **Figure 3B** – upper left panel. **Table 5** shows this pattern explicitly and demonstrates the moderation effect AA has on EV: under the *Hunger* condition (but not the *Non-Hunger* condition) AA levels moderated the probability to choose the lottery offer. In other words, as AA levels decrease, when subjects are objectively hungrier, their propensity to choose the lottery increases.

**Table 5:** Simple slopes analysis – AA levels during the hunger session moderate the effect of EV on lottery choices.

				95% CI	
AA level	EV coefficient	*SE*	*Z*	ll	ul
-2SD	0.169348	0.023793	7.12^∗∗∗^	0.122714	0.215982
-1SD	0.145402	0.015777	9.22^∗∗∗^	0.114479	0.176325
Mean	0.121455	0.009565	12.7^∗∗∗^	0.102708	0.140202
+1SD	0.097508	0.009686	10.07^∗∗∗^	0.078524	0.116493
+2SD	0.073562	0.015998	4.6^∗∗∗^	0.042207	0.104916

*^∗∗∗^p < 0.001.*

In the same logistic regression, we also examined the effect of subjective VAS scores (as reported by our subjects) on risk preferences. Strikingly, we did not find any significant effects of VAS scores on the tendency to choose the lottery option in either reward type and in either session (**Table 4**). This strengthens the notion that subjective reports of hunger levels are not an accurate or reliable indicator of the effect that hunger levels have on risk preferences. Moreover, we found that *Condition* itself did not significantly affect risk preferences (money: *B* = 0.13, *p* = 0.5; food: 0.08, *p* = 0.72), indicating that instructing subjects to refrain from eating does not guarantee that they will do so. In fact, even if all subject did in fact fast as we requested, their objective hunger levels vary drastically, preventing a general main effect of *Condition* to reach significance.

Following these analyses, we also examined separately the effect of AA levels and VAS scores on risk preferences in the monetary domain. We found that the model with AA levels (without VAS scores, alongside with *Condition*, EV, and their interactions) replicates the results of the full model. That is, we found a main effect for EV (*B* = 0.12, *Z* = 13.43, *p* < 0.000), an EV by AA interaction (*B* = -0.08, *Z* = -2.38, *p* < 0.05), and most importantly a significant three-way interaction between *Condition*, EV, and AA (*B* = 0.08, *Z* = 2.03, *p* < 0.05). However, when we replaced AA scores with VAS scores, we found only an EV effect (*B* = 0.12, *Z* = 12.52, *p* < 0.000), and no three-way interaction (*B* = 0.01, *Z* = 0.94, n.s.). VAS main effect was not significant as well, as any other VAS-related effect (all *p* > 0.6).

### Ultimatum Game

In addition to examining risk preferences, we examined how hunger levels influence social decision-making in the same subjects. Specifically, we examined how hunger levels affect subjects’ propensity to accept unfair offers in the ultimatum game.

**FIGURE 4:**
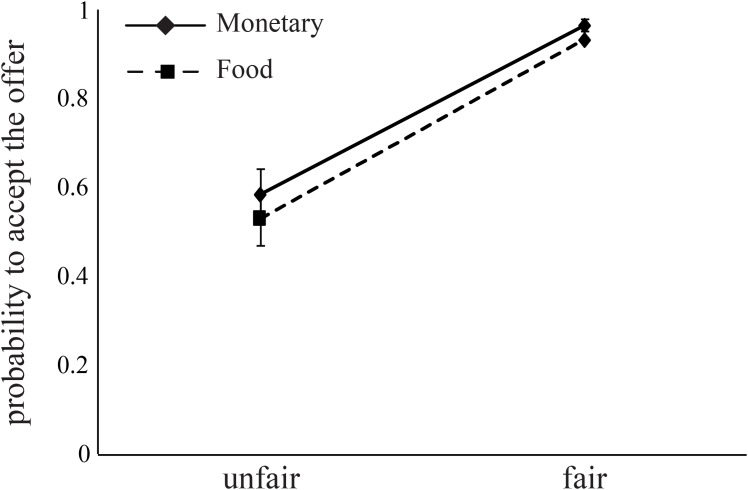
Subjects decline unfair offers for both food and money. Fair offers (8 or 10 out of 20, NIS or chocolates) were accepted by subjects at higher rates than unfair offers (2 or 4 out of 20). Error bars indicate standard error of the mean.

The probability to accept an offer increased as the offer changed from unfair (mean acceptance rate: *M* = 0.56, *SD* = 0.06) to fair (mean acceptance rate: *M* = 0.95, *SD* = 0.01, **Figure 4**). This effect is evident in both monetary (*B* = 2.99, *p* < 0.0001) and food (*B* = 3.61, *p* < 0.0001) offers (**Table 6**). Importantly, we found a significant interaction between FI, AA levels, and *Condition* for monetary offers (*B* = 7.45, *p* < 0.01, **Table 6**), a marginally significant main effect of AA on food offers (*B* = 2.89, *p* = 0.055), and a close to significant interaction between AA and *Condition* (*B* = -3.13, *p* = 0.068) for food offers.

**Table 6:** Effect of fairness (FI), condition (hunger/non-hunger), subjective hunger measure (VAS), and objective hunger measure (AA) on acceptance rates in an ultimatum game.

	Reward type					
	Monetary			Food		
Parameters	*B*	*SE*^a^	*Z*	*B*	*SE*^a^	*Z*
FI	2.99	0.63	4.72^∗∗∗^	3.61	0.91	3.97^∗∗∗^
Condition	0.34	0.24	1.42	0.09	0.24	0.37
Condition × FI	0.74	0.73	1	-1.08	1.17	-0.93
AA	0.4	1.52	0.26	2.98	1.58	1.89^#^
FI × AA	-2.33	2.34	-1	-3.73	5.29	-0.7
Condition × AA	-0.47	1.56	-0.3	-3.13	1.72	-1.82^#^
Condition × FI × AA	7.45	2.32	3.21^∗∗^	6.89	5.59	1.23
VAS	-0.14	0.26	-0.54	0.29	0.29	1.03
FI × VAS	0.34	0.57	0.6	0.69	0.53	1.28
Condition × VAS	-0.15	0.31	-0.49	-0.37	0.32	-1.16
Condition × FI × VAS	0.03	0.62	0.05	-0.33	0.61	-0.54

*^a^Robust Std. Err. (errors clustered by subject); ^∗^p < 0.05, ^∗∗^p < 0.01, ^∗∗∗^p < 0.0001, ^#^p < 0.1.*

**FIGURE 5:**
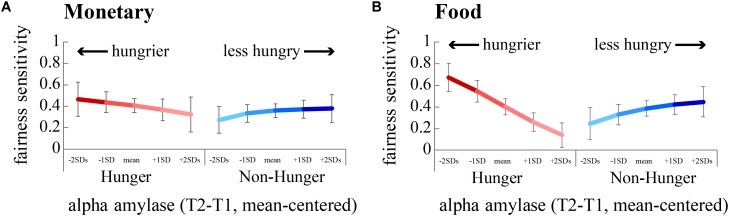
Sensitivity to fairness is modulated by hunger levels. We estimated subjects’ sensitivity to fairness by dividing the acceptance rate for fair offers by unfair offers. A greater difference indicates a greater sensitivity to fairness. The hungriest subjects (negative standard deviations from the mean of AA, dark red lines) were most sensitive to fairness, by rejecting more unfair offers than fair offers. **(A)** Fairness sensitivity to monetary offers. **(B)** Fairness sensitivity to food offers.

We focused on subjects’ sensitivity to fairness, i.e., the propensity to accept a fair offer vs. the propensity to accept an unfair offer, and how this sensitivity was affected by hunger. Running two separate regression models for monetary rewards – one for the *Hunger* condition and one for the *Non-hunger* condition – revealed that there is a significant two-way interaction (between AA and sensitivity to fairness) within the *Hunger* condition (*B* = 5.12, *p* < 0.01), and a close to significant interaction in the *Non-Hunger* condition (*B* = 3.16, *p* = 0.06; **Figure 5A**). On the one hand, under the *Hunger* condition, as subjects become objectively hungrier (lower levels of AA), sensitivity to fairness increased (the difference in acceptance rates grows from 0.32 to 0.46). On the other hand, in the *Non-Hunger* condition, we observe an opposite effect – as subjects became objectively hungrier (lower levels of AA) the fairness sensitivity decreased (from 0.37 to 0.27). A very similar pattern was evident for food offers (**Figure 5B**). Much like the risk task, these findings demonstrate that AA levels moderate subjects’ choices, albeit in a more complex manner. It appears that moderate hunger, but not extreme hunger, causes subjects to become less vindictive.

Finally, in the same logistic regression we also examined whether subjective reports of hunger levels (VAS scores) influence subjects’ choices to accept unfair offers. As can be seen in **Table 6**, we did not find any significant effects when using VAS scores in any of the experimental conditions for either reward type. This reinforces our claim that subjective reports are not a suitable proxy for assessing hunger levels and how they might affect choice, while using an objective physiological marker yields a more accurate indication and reveals the complexity of the effect of hunger levels on choice.

### Economic Inconsistency

One of the fundamental axioms in neoclassical economics is choice consistency, or the GARP. Specifically, according to expected utility theory, subjects need to demonstrate consistency in their choices in order to assume that they are rational agents that maximize a utility function (Samuelson, 1938; Houthakker, 1950). Because this is a basic requirement from a rational agent we were interested to examine if this behavior is affected by changing hunger levels. In order to do so, subjects completed a task aimed to estimate their level of consistency. We used a well-known task developed and described in Choi et al. (2007). Subjects were requested to allocate an endowment of tokens (using a mouse curser) between two lotteries with equal probabilities (50–50% chance of winning) corresponding to the *X-* and *Y*-axes on a two-dimensional graph. Based on subjects’ allocations we estimated the level of economic inconsistency (GARP violations) for each subject, using three well-known indices taken from the economic literature – Afriat index (Afriat, 1973), Varian index (Varian, 1990), and Houtman–Maks index (Houtman and Maks, 1985). We analyzed the effect of hunger levels on subjects’ consistency levels using OLS regressions. However, we did not find any significant effects of either *Condition*, AA levels, or VAS scores on consistency levels. This suggests that choice consistency as measured in our paradigm is relatively robust to this degree of change in hunger levels.

### Correlation Across Tasks

**Table 7** describes the correlations between subjects’ behaviors across risk, ultimatum game, and economic inconsistency tasks. As can be seen, the correlation within tasks is high – suggesting that there is high consistency across subjects in risk and social preferences, and to a lesser degree in economic inconsistency. However, our sample shows that the correlations between the tasks are extremely low, suggesting that there are no direct links between these tasks.

**Table 7:** Average performance in tasks and correlation across tasks: propensity to choose lottery on the risk task, propensity to accept the offer on the ultimatum game (UG), Varian index on the economic inconsistency (EI) task.

Task	Reward type	Condition	Mean (*SD*)	#	(1)	(2)	(3)	(4)	(5)	(6)	(7)	(8)	(9)	(10)
Risk	Food	H	0.32 (0.18)	(1)	–									
		NH	0.32 (0.20)	(2)	0.67^∗∗∗^	–								
	Money	H	0.32 (0.16)	(3)	0.79^∗∗∗^	0.61^∗∗∗^	–							
		NH	0.31 (0.16)	(4)	0.50^∗∗∗^	0.81^∗∗∗^	0.68^∗∗^	–						
UG	Food	H	0.72 (0.29)	(5)	0.16	-0.01	0.10	-0.12	–					
		NH	0.73 (0.28)	(6)	0.08	-0.13	0.10	-0.13	0.73^∗∗∗^	–				
	Money	H	0.75 (0.26)	(7)	0.12	-0.15	0.06	-0.23	0.60^∗∗∗^	0.57^∗∗∗^	–			
		NH	0.81 (0.24)	(8)	0.12	-0.05	0.18	-0.05	0.43^∗∗^	0.65^∗∗∗^	0.61^∗∗∗^	–		
EI	Money	H	0.05 (0.15)	(9)	-0.06	0.28	0.28	0.35^∗^	0.17	0.07	0.17	0.21	–	
		NH	0.07 (0.18)	(10)	-0.02	0.25	0.15	0.28	-0.01	-0.13	-0.03	0.08	0.43^∗∗^	–

*H, hunger session; NH, non-hunger session. ^∗^p < 0.05, ^∗∗^p < 0.01, ^∗∗∗^p < 0.001.*

## Discussion

In the current study, we set out to explore the ways in which hunger affects a number of human economic behaviors: risk-taking, social preferences, and consistency. Notwithstanding, we had another major aim in mind – introduce a new method to objectively and reliably measure levels of hunger. Using three separate behavioral paradigms, we found that hunger tips subjects’ risk preferences toward more risk-seeking, modulates their sensitivity to fairness, but evidently has no detectable effects on consistency of economic choices. More importantly, we show that our objective measure of hunger moderates the behavioral effects of hunger, while the commonly used subjective self-report measure fails to do so.

Our first finding, that hungrier subjects are more inclined to choose the lottery option over a safe amount of money, is in accordance with some previous work (Symmonds et al., 2010; Levy et al., 2013), that showed a greater tendency to be less risk-averse when hungry. These previous findings and our current results are in line with the notion that organisms tend to become less risk averse (and even risk seeking) under extreme hunger conditions because they need to take more risks in order to find food for survival. Presumably, although our subjects were not starving, this tendency of hungry individuals to become less risk averse is evidence of how evolutionary pressure from the past is still influencing our behavior. However, as mentioned above, there are contradictory findings as for the direction of the effect of hunger on risky behaviors. While some studies report an increase in risk-seeking behavior under deprivation conditions, others find the opposite effect, with subjects becoming more risk-averse as resources become scarce. A possible explanation to the discrepancy in results may arise from individual differences across the measured population, with different members of a species reacting differently to the same changes in environment. This phenomenon was demonstrated in a previous work, in which the authors found that hunger has a converging effect on a population – individuals who were highly risk-averse when satiated became less so when hungry, while risk-seeking individuals became more risk-averse (Levy et al., 2013). Another possible explanation for the discrepancy is that different studies manipulate hunger to varying degrees, which suggests that the modulation of risk preferences depends on the severity of deprivation. Furthermore, several previous studies that showed contradicting findings relied on self-reports for estimating hunger levels. As we clearly demonstrated in the current study, hunger levels as estimated using self-reports were not associated with any of our choice tasks, and they were not a reliable indication of the “true” hunger levels but rather a mix of hunger levels, desirability bias, and experimental demand effects. Moreover, animal studies showed that not all hungry animals (even to the same extent) behave the same way. Only animals that were under a negative budget rate environment (i.e., the rate of food arrival/consumption will not be enough for the animal to survive) demonstrated risk-seeking behavior while animals with a positive budget rate did not (Caraco et al., 1980). Interestingly, we find a difference in risk preferences only under the Hunger condition. This suggests that only a true state of hunger influences risk attitudes. That is, when subjects were in the Non-Hunger session, although differences in AA between subjects were present, they are not as hungry as they are in the Hunger session. Only when faced with substantial hunger, as is experienced in the Hunger session, subjects’ attitude toward risk change – and this change is moderated by the individual differences in the *level* of physiological hunger, such that the hungriest subjects exhibit the biggest risk-seeking behavior. Our objective measure of the physiological hunger state was able to capture the subject-by-subject variability in hunger levels and therefore serve as a good proxy for the effect that individual hunger levels have on individual risk preferences. In any way, careful and systematic study of humans as well as other animals will be needed to tease apart the contribution of environment and individual differences. Notwithstanding, the use of physiological tools to measure the objective levels of hunger would be crucial for such studies, and as we show here, the AA enzyme is a prime candidate to study the mediating effect of hunger on risk preferences.

Our second finding is that subjects under extreme physiological states – either very hungry or very not hungry – exhibit greater sensitivity to fairness, by rejecting more unfair offers. A moderate internal state, such as a mild hunger, however, renders people less vindictive, with similar acceptance rates for fair and unfair offers. Much like studies of hunger and risk, previous studies that examined the effect of deprivation on social behavior struggled to reach a consensus – it appears that under various conditions and states, and for different individuals, deprivation causes different and opposite behaviors. For example, one study found hungry subjects to be less inclined to donate to charity, i.e., deprivation reduced social preferences (Briers et al., 2006). Another study, however, reported a negative association between consumption of sugary drinks with support for social welfare, i.e., deprivation was actually linked to increased social preferences (Aaroe and Petersen, 2013). Note that the latter study found no change in actual sharing behavior, only in the stated self-reported behavior, demonstrating yet again the feebleness of subjective measures. Other studies have examined the effect of stress on subjects’ prosocial behavior, and found that stress increases it (Buchanan and Preston, 2014). However, at least one study reports a temporal dependency for this effect, showing that only 90 min after exposure to acute stress subjects exhibit an increase in acceptance rates, but not immediately after (Vinkers et al., 2013). Furthermore, it seems that the prosocial effect of stress is dependent upon the affect it evokes – individuals who perceive the stress as positive (a challenge) become more altruistic, whereas individuals who perceive it as a threat become more selfish (Buchanan and Preston, 2014). We therefore propose, that the u-shaped association between levels of hunger and levels of fairness-sensitivity that we report here hints to the dynamic nature of hunger effects on prosocial preferences, similar to that of stress. That is, under mild deprivation human subjects tend to become less sensitive to fairness (increasing prosocial behavior), whereas under extreme hunger they demonstrate higher degrees of vindictiveness. It is important to note that higher acceptance rates of unfair offers may represent either prosocial behavior (acting altruistically toward the proposing partner) or an increase in selfishness (acting to increase self-gain) (Mancini et al., 2011). Our paradigm cannot distinguish between the two motivations, but it may be that the decrease in sensitivity to fairness we observe in extremely hungry subjects stems in fact from a self-preserving motivation, and not a change in social preference. Further research will be needed to specifically address this hypothesis.

In our third and final behavioral task, we could not identify an effect of hunger on choice consistency. From this null result, we must not make any general statements that hunger levels do not have an effect on choice consistency. All we can conclude is that under 12 h of fasting, using the GARP task and our measurement tool of hunger levels, we could not detect any changes in choice inconsistency as a function of hunger levels. Strengthening this possibility is a recent study that showed that marmosets display *more* consistent behavior as they become hungrier (Yamada, 2017). Hence, because our subjects demonstrated a relatively high level of consistency throughout the task and in both conditions, our failure to detect differences in consistency as a function of hunger levels may be due to a ceiling effect. It is also possible that in order to detect changes in the level of choice consistency subjects need to be a lot hungrier. We plan to examine this in future studies.

The most important finding of the current paper is that only an objective measurement of hunger levels, but not subjective reports, could accurately measure subjects’ hunger levels and serve as a reliable indication of how subject-by-subject variability in hunger levels influences choice behavior. Moreover, we demonstrated that an objective reliable measurement of hunger levels is crucial for the understanding of the complex effects hunger has on choice behavior. We show that looking at group averages alone or examining only the main effect of the manipulation (which was not significant) might lead to wrong conclusions regarding the effect of hunger on choice behavior.

Any conclusion drawn from a study first and foremost depends on the reliability of the measures it employs. Self-reports, common in social sciences, were often found to be biased by personality traits such as social-desirability or social-approval biases (Podsakoff et al., 2003). For example, subjects report inaccurately their height and weight (Gorber et al., 2007), their dietary intake (Freedman et al., 2014), as well as their attitudes and beliefs about others (Greenwald et al., 1998). We therefore consider subjective hunger ratings as malleable and imprecise. Although self-report measures may have value in and of themselves for studying the effects of a *psychological* state of hunger on behavior, which cannot be studied using physiological markers, we argue that their contamination with non-relevant information renders them unsuitable for our study. That is not to say that all self-report measures are wrong, but that their implementation must be highly controlled, in order to preclude subjects from adjusting their response to appease the experimenters. Furthermore, when studying the effects of *physiological* hunger on behavior, as we are here, the use of subjective tools is tricky at best. In our current work, we present both the need for an objective measure of an organism’s internal state, and put forward the novel use of a salivary enzyme called alpha-amylase as such a measure. Naturally, the most direct, accurate, and straightforward way of physiologically measuring hunger levels is via a blood sample, as have several previous studies done (Symmonds et al., 2010; Aaroe and Petersen, 2013). Some of these studies focused on glucose and/or insulin levels. However, in healthy humans the blood-level changes of these markers are rapid, happening in a relatively short time scale (Dinneen, 1997), and do not change very much due to the natural process of homeostasis (Anderson et al., 1967; Alzaid et al., 1994; Cohen, 2006). Hence, glucose or insulin levels are not good predictors of hunger levels after 12 h of fasting (when, in general, glucose levels are back to baseline) but rather an indication of the current number of calories entered into the body, and how fast can the body adjust to the rise in glucose (as is done in a glucose tolerance test, (Ernsberger and Koletsky, 2012; Zhou et al., 2016). Furthermore, there are contradicting results regarding the effect of sucrose levels on decision-making (Molden et al., 2012) (for a review, see Orquin and Kurzban (2016)].

An alternative measure is ghrelin and/or leptin, both of which are regulatory hormones taking part in the hunger/satiety cycle (Klok et al., 2007), which were shown to be relatively reliable indications for current hunger levels (ghrelin) and long-term fat storage (leptin) (Klok et al., 2007), as well as moderate risk preferences to some extent (Symmonds et al., 2010). However, although blood measures are arguably most accurate in assessing an organism’s internal state, including a blood draw procedure in an experiment may have undesirable consequences. First, it can potentially bias the selection of subjects, by excluding individuals with needle-anxiety. Second, drawing blood may have a stressogenic effect on subjects, which may alter their behavior (Buckert et al., 2014; Bendahan et al., 2017). Third, the procedural implications are plentiful: drawing blood requires skilled personnel, expansive equipment, and special requirements from ethics committees. On the other hand, using saliva samples is relatively cheap, easy-to-use, does not induce stress, and minimizes the selection bias.

## Conclusion

Our finding hinders the traditional and wide use of the visual analog scale as a measure of individuals’ hunger levels, and suggests that it is unreliable and may lead researchers to wrong conclusions about the manner in which hunger affects behavior. We strongly believe that future researchers interested in the interaction of physiology with psychology should employ objective physiological tools, such as the AA enzyme. The method we introduce here is perfectly tailored for studies in behavioral economics and marketing, due to its ease of use and high applicability. Especially in a field of study of such abundant implications and interest, alongside highly controversial and contradicting finding thus far, researchers should employ the most meticulous and precise methods as possible.

## Author Contributions

DL designed the behavioral aspects of the experiment. MS-S designed the biological aspects of the experiment. MS-S and AS collected the data. MS-S, AS, and TS analyzed the data. MS-S, AS, TS, and DL wrote and edited the manuscript.

## Conflict of Interest Statement

The authors declare that the research was conducted in the absence of any commercial or financial relationships that could be construed as a potential conflict of interest. The reviewer LS and handling Editor declared their shared affiliation.
